# Insomnia and Insomnia-Related Care in the Department of Veterans Affairs: An Electronic Health Record Analysis

**DOI:** 10.3390/ijerph18168573

**Published:** 2021-08-13

**Authors:** Adam D. Bramoweth, Caitlan A. Tighe, Gregory S. Berlin

**Affiliations:** 1 VISN 4 Mental Illness Research, Education and Clinical Center, VA Pittsburgh Healthcare System, Research Office Building (151RU/MIRECC), University Drive, Pittsburgh, PA 15240, USA; Caitlan.Tighe@va.gov; 2 Center for Health Equity Research and Promotion, VA Pittsburgh Healthcare System, Research Office Building (151C), University Drive, Pittsburgh, PA 15240, USA; 3 VA Connecticut Healthcare System, 950 Campbell Avenue (116B), West Haven, CT 06516, USA; Gregory.Berlin@va.gov; 4 Department of Psychiatry, Yale University School of Medicine, 300 George Street, New Haven, CT 06511, USA

**Keywords:** insomnia, cognitive behavioral therapy for insomnia, sedatives and hypnotics, veterans, veterans affairs

## Abstract

The objective was to examine insomnia and insomnia-related care within a regional network of Department of Veterans Affairs (VA) facilities since the VA roll-out of cognitive behavioral therapy for insomnia (CBT-I) in 2011. A retrospective analysis of VA electronic health records (EHR) data from 2011 to 2019 was conducted. The annual and overall prevalence of four insomnia indicators was measured: diagnoses, medications, consultations for assessment/treatment, and participation in CBT-I. Also examined were sociodemographic and clinical differences among veterans with and without an insomnia indicator, as well as differences among the four individual insomnia indicators. The sample included 439,887 veterans, with 17% identified by one of the four indicators; medications was most common (15%), followed by diagnoses (6%), consults (1.5%), and CBT-I (0.6%). Trends over time included increasing yearly rates for diagnoses, consults, and CBT-I, and decreasing rates for medications. Significant differences were identified between the sociodemographic and clinical variables across indicators. An evaluation of a large sample of veterans identified that prescription sleep medications remain the best way to identify veterans with insomnia. Furthermore, insomnia continues to be under-diagnosed, per VA EHR data, which may have implications for treatment consistent with clinical practice guidelines and may negatively impact veteran health.

## 1. Introduction

Insomnia—broadly defined as difficulty initiating or maintaining sleep and dissatisfaction with sleep quantity or quality—is common in adults and is associated with adverse physical and mental health outcomes [1,2,3,4]. In the general population, about 30% of adults report at least one symptom of insomnia (e.g., difficulty falling asleep/staying asleep) and an estimated 6–10% meet diagnostic criteria for insomnia disorder (symptoms ≥3 months duration, ≥3 days per week, daytime impairment [1,5]). The prevalence of insomnia is estimated to be even higher in United States (US) military service members and veterans. Despite its high prevalence and known adverse health impacts, insomnia remains under-diagnosed and under-treated in clinical settings [6,7]. 

Survey studies suggest that upwards of half of US Military service members and veterans experience clinically significant insomnia symptoms (see US Department of Veterans Affairs (VA)/Department of Defense (DOD) guidelines for review [8]). Notably, a recent study of post-9/11 veterans found that 57.2% scored ≥11 (clinical cutoff) on the Insomnia Severity Index and 40.5% scored ≥15 (moderate-severe cutoff), both scores indicative of increased risk for a diagnosis of insomnia disorder [9]. In contrast, administrative studies of VA electronic health records (EHR) estimate insomnia prevalence rates from 0.2% to 3.5% [6,7]. Though a 650% relative increase in the age-adjusted prevalence of insomnia was observed in veterans utilizing VA services between fiscal years 2000 and 2010 (from 0.2% to 1.5% [6]), there remains a notable discrepancy between reports of clinically significant insomnia symptoms [9] and expected diagnoses of insomnia in the medical record (7.4–11.8%; [7,10,11]).

An additional discrepancy arises with regard to insomnia diagnosis and treatment. Per the VA/DOD Clinical Practice Guidelines, as well as recommendations from the American Academy of Sleep Medicine, the American College of Physicians, and the National Institutes of Health, the first-line treatment of insomnia for adults is cognitive behavioral therapy for insomnia (CBT-I) [8,12]. A multi-component evidence-based psychotherapy consisting of cognitive therapy, behavior change strategies, and educational components, CBT-I not only improves insomnia severity and global sleep outcomes, but also improves comorbid medical and psychiatric outcomes in adults [13,14]. Despite these recommendations and support from numerous clinical trials, individuals with an insomnia complaint are significantly more likely to receive an insomnia medication instead of being referred for, or receiving, CBT-I [11].

Notably, VA has invested substantially in the dissemination of CBT-I. In 2011, the VA Office of Mental Health and Suicide Prevention (formerly Office of Mental Health Services) rolled out provider training for CBT-I as part of the evidence-based psychotherapy (EBP) training program, and there are now over 1000 trained CBT-I clinicians [15]. Evaluation data from the rollout have established that VA clinicians can be effectively trained to competently deliver CBT-I and that veterans who complete treatment tend to experience significant improvements in their insomnia severity [16,17]. However, much less is known about how insomnia-related care processes have changed since the rollout.

The overarching goal of this analysis was to examine insomnia and insomnia-related care broadly within a network of VA facilities in the mid-Atlantic region of the US (Veterans Integrated Service Network 4, VISN 4) since the VA roll-out of CBT-I in 2011. To do this, we conducted a retrospective analysis of VA EHR to evaluate four insomnia-related care indicators from fiscal year (FY, 1 October–30 September) 2011 through 2019: (1) insomnia diagnoses, (2) prescriptions of insomnia medication, (3) consultations for insomnia assessment/treatment, and (4) treatment with CBT-I. We also examined select sociodemographic and clinical groups with and without an insomnia indicator.

## 2. Materials and Methods

### 2.1. Study Population and Procedures

We analyzed veteran data obtained from the VA Corporate Data Warehouse (CDW), a repository of data from clinical and administrative VA systems. The sample included veterans who had ≥1 outpatient clinical encounter in VISN 4, a regional network of VA facilities in mid-Atlantic region of the US (Pennsylvania, Delaware, and parts of Ohio, West Virginia, New York, and New Jersey). The data is from clinical encounters between 10/01/2011 and 09/30/2019 (FY2011–2019). Veterans who had ≥1 outpatient clinical encounter in VISN 4 during the study period but received the majority of their care at VA facilities outside of VISN 4 were excluded for that particular fiscal year. This study was approved by the VA Pittsburgh Healthcare System (VAPHS) Institutional Review Board (protocol number PRO00001980; approved on 11 August 2017).

Insomnia diagnosis. Insomnia diagnoses were identified with corresponding outpatient International Classification of Diseases—Ninth Revision (ICD-9: 307.42; 327.00, 327.01, 327.02, 327.09; 780.51, 780.52) and International Classification of Diseases—Tenth Revision (ICD-10: F51.01, F51.03, F51.04, F51.05, F51.09; G47.00, G47.01, G47.09) codes. An insomnia diagnosis was determined as present when ≥2 ICD codes from the above list occurred >30 days apart but within 390 days of each other (the equivalent of 13 30-day months; 30/390 criteria). Similar to the approach by Alexander and colleagues [6], this approach limits the detection of rule-out diagnoses and extends beyond one calendar year to account for variability in annual appointment scheduling. 

Insomnia medication. Insomnia medications were identified using outpatient pharmacy records and were indicated by filled prescriptions of >15 pills of any of the following medications: doxepin (3 mg, 6 mg), eszopiclone (1 mg, 2 mg, 3 mg), ramelteon (8 mg), suvorexant (10 mg, 15 mg, 20 mg), temazepam (7.5 mg, 15 mg, 22.5 mg, 30 mg), trazodone (50 mg, 100 mg, 150 mg), zaleplon (5 mg, 10 mg), zolpidem (5 mg, 6.25 mg, 10 mg, 12.5 mg), zolpidem tartrate (1.75 mg, 5 mg (spray and tablet), 6.25 mg, 10 mg, 12.5 mg). These medications and dosages were selected based on a combination of clinical practice guidelines [18], medication indications for the treatment of insomnia (on- and off-label), and common clinical practice. We did not include medications such as most benzodiazepines and some sedating anti-depressants and anti-psychotics, that are used to treat insomnia and are commonly used to treat other disorders, to limit over-estimation of insomnia-related medication utilization. Thus, within our sample, the use of insomnia medications as an insomnia indicator will likely underestimate the true prevalence of medication use intended to treat insomnia. 

Consults for insomnia assessment/treatment. Consults refer to the intra- or inter-facility process of referring to a specific type of care using the VA EHR. Consults are created by each facility and are not consistent across VAMCs. For the current analyses, consults for insomnia treatment were operationalized as the frequency of consults identified in the EHR that included the stem “insom” for the provisional diagnosis, a required field representing the reason for consultation request, or in the service/clinic requested by a healthcare provider, another required field. 

CBT-I delivery. CBT-I treatment delivery was identified using VA evidence-based psychotherapy (EBP) note templates that are available in the EHR. The VA CBT-I EBP templates are structured documentation templates that correspond with the core components of each treatment session (i.e., evaluation, initial treatment session, middle treatment session, final session) and help to support fidelity in the delivery of CBT-I. Only CBT-I-templated notes are reported as these are standardized and recommended per the VA CBT-I EBP program. Other types of progress notes (e.g., notes that are not templated) that reflect the delivery of CBT-I were not collected. The annual frequencies of individuals who received ≥1 CBT-I templated note are reported. 

Sociodemographic variables. Veterans were grouped by sex, race, age, and zip code per the data available in the EHR. Sex was either male or female. Race was White, Black, or Other (i.e., all other individual races (American Indian or Alaska Native; Asian; Native Hawaiian or Other Pacific Islander), mixed race, declined, or unknown). Age was determined during FY2011 and dichotomized as younger than 50 or 50 years and older. Veterans were also grouped by decile (20–90+) with the 60–69 years group being the largest. Veterans were grouped according to the rurality of the county of their home address according to the 2013 Rural-Urban Continuum Codes (RUCC; [19]). Veterans were classified into two groups: metropolitan/urban, metropolitan-adjacent counties (metro) and urban, non-metropolitan adjacent/rural counties (non-metro). 

Clinical variables. Mental health disorder and substance use disorder variables were derived from ICD-9/10 codes using the same criteria as the insomnia diagnoses (i.e., 30/390 criteria). The mental health disorder variable did not include insomnia ICD codes used to operationally define the insomnia diagnosis variable and also did not include substance use disorder ICD-9/10 codes. The substance use disorder variable did not include nicotine-related ICD-9/10 codes. While the goal was to keep clinical categories broad, distinguishing mental health and substance use has clinical relevance. Mental health disorders are some of the most common comorbid disorders for individuals with insomnia; however, identifying specific disorders was beyond the goal of this initial evaluation. Medical comorbidities were calculated for this sample by body system (e.g., respiratory, endocrine, circulatory, digestive, etc.). However, due to the high rate of body systems impacted among veterans (average of 9/veteran [20]), for the current set of broad analyses, the body system/medical comorbidity groups were excluded but will be utilized in future analyses with a focus on more specific factors that impact insomnia-related care. 

### 2.2. Statistical Analysis 

Analyses were completed using MedCalc Statistical Software (v19.2.6; [21]). To evaluate insomnia and insomnia-related care over the study period, we examined the annual and overall (FY2011–2019) prevalence of insomnia diagnoses (ICD), insomnia medications (MED), number of consultations for insomnia (CONS), and counts of CBT-I treatment received (CBTI). We also examined select sociodemographic and clinical differences among veterans with and without an insomnia indicator (any of ICD, MED, CONS, CBTI) as well as differences among the individual insomnia indicators (ICD, MED, CONS, CBTI). This was done first as a comparison of proportions between the sociodemographic and clinical groups (N-1 Chi-squared test, 95% confidence intervals [CI]) and also calculating relative risk (RR and 95% CI). Analyses were completed for the overall sample (any insomnia vs. no insomnia) and within each insomnia indicator (yes/no for ICD, MED, CONS, CBTI limiting sample to those with any insomnia indicator).

## 3. Results

The study sample included 439,887 veterans in VISN 4 (FY2011–2019), which includes the following VA Medical Centers and Health Care Systems: Altoona, Butler, Coatesville, Erie, Lebanon, Pittsburgh, Philadelphia, and Wilkes-Barre in Pennsylvania and Wilmington, Delaware. The sample was 94% men and 6% women with an average age of 59.85 (SD = 17.92, range 20–90+). In terms of veteran race, 78.4% were White, 12.8% were Black, and 8.8% were classified as Other. 

Veterans were identified by the four insomnia indicators—insomnia diagnoses (ICD), prescriptions of an insomnia medication (MED), (3) consultations for insomnia assessment/treatment (CONS), and treatment with CBT-I (CBTI)—and 17% of the sample were identified by at least one of the four insomnia indicators (*n* = 74,966). Approximately 6% (*n* = 26,375) had an insomnia diagnosis and 15% (*n* = 64,036) were prescribed an insomnia medication at some point during the study period (FY2011–FY2019). See Table 1 for prevalence and yearly counts for each insomnia indicator. The annual prevalence of insomnia diagnoses ranged from 1.96% in FY2011 to 3.34% in FY2019. The annual prevalence of an insomnia diagnosis increased each year over the course of the study period with the exception of from FY2015 to FY2016; this decrease in insomnia diagnoses from FY2015 to FY2016 could be an artifact in the EHR related to the transition from ICD-9 to ICD-10. The annual prevalence of insomnia medications decreased over the study period, ranging from a high of 9.08% in FY2011 to 7.32% in FY2019. Only 1.5% of the sample (*n* = 6627) received a consultation for the assessment/treatment of insomnia and there was more than a four-fold increase in the annual frequency of consults, with the fewest at the beginning of the study period (*n* = 338, FY2011) and the most at the end (*n* = 1392, FY2019). Less than 1% (0.55%) of veterans in the sample engaged in CBT-I (*n* = 2441), although these data were only available for part of the study period, FY2015–2019, as CBT-I-templated notes were not available in the VA EHR until FY2015.

When evaluating age deciles (Table 2), veterans aged <60 years had significantly higher proportions and relative risk compared to the 60–69 years group and those ≥70 years (70–79, 80–89, 90+) had significantly lower proportions of any insomnia indicator and relative risk compared to the 60–69 years group. See Table 3 for the comparisons of the sociodemographic and clinical groups. There were significant differences in proportions and relative risk analyses for veterans with any insomnia indicator. Women (vs. men), Black (vs. White), younger (vs. older), and veterans with mental health and/or substance use disorders (vs. not), all had significantly greater proportions of any insomnia as well as greater relative risk. Veterans classified with Other race (vs. White) and those who live in a non-metro county (vs. metro county) had a significantly lower proportion of any insomnia indicator and lower relative risk. The largest difference in proportion and relative risk occurred among veterans with or without mental health disorders. Veterans with mental health disorders had 25% greater proportion (32.6% vs. 7.3%) for the presence of any insomnia indicator compared with those without mental health disorders. Moreover, they were nearly 450% more likely to have any insomnia indicator compared with veterans without a mental health disorder.

We also evaluated each sociodemographic and clinical variable for differences in proportion and relative risk for each individual insomnia indicator, limiting the sample to those with any insomnia indicator (*n* = 74,966). For veterans with an insomnia diagnosis, only those who lived in a metro county (vs. non-metro) had a greater proportion and relative risk. Veterans who were female, Black, younger, and with a mental health or substance use disorder had lower proportion and relative risk compared to their respective group counterparts. Veterans within the Other race group had no significant difference (vs. White race). The largest proportion difference was among veterans with or without a mental health disorder (33.4% vs. 40.3%); veterans with a mental health disorder were 21% less likely to have an insomnia diagnosis (RR = 0.83).

For veterans with prescription insomnia medication, females and those with a mental health or substance use disorder all had a greater proportion and relative risk. Veterans with a mental health disorder again had the largest proportion difference (88.4% vs. 76.9%) and were 15% more likely to be prescribed an insomnia medication (RR = 1.15). Veterans who were younger had lower proportion and relative risk. There were no significant differences for veterans who were Black or Other race and those who lived in non-metro counties. 

For veterans who received a consultation for the assessment/treatment of insomnia, female, younger, and Black veterans had greater proportion and relative risk. Those veterans with a mental health disorder, substance use disorder, and who lived in a non-metro country had lower proportion and relative risk. The largest proportion difference was younger (vs. older) veterans (11.9% vs. 7.0%) who were 72% more likely to receive a consultation for insomnia (RR = 1.72). Veterans classified as Other race had no significant difference (vs. White race). 

Lastly, treatment with CBT-I was analyzed. Similar to the consultation analyses, veterans who were female, Black and Other race, and younger had greater proportion and relative risk for engaging in treatment with CBT-I. Those with a mental health disorder, substance use disorder, and lived in non-metro counties had lower proportion and relative risk. The largest proportion difference was for veterans classified as Black race (5.1% vs. 2.8%) and those in non-metro counties (1.0% vs. 3.3%); veterans who lived in non-metro counties were 345% less likely to engage in CBT-I vs. their metro county-living counterparts (RR = 0.29).

## 4. Discussion

This manuscript represents an important step toward understanding and characterizing insomnia and insomnia-related care in the VA based on data acquired from the EHR. Four insomnia indicators—insomnia diagnoses (ICD), sleep medications (MED), consultations for assessment/treatment (CONS), and delivery of CBT-I (CBTI)—were evaluated among veterans in VA VISN 4 from FY2011–2019, with sociodemographic and clinical comparisons conducted for each indicator. The findings from this large veteran dataset are consistent with previous studies in suggesting that the prevalence of insomnia (defined by ICD codes) is increasing, yet remains lower than estimates from studies using other methods and data sources (e.g., surveys, self-report). Findings also suggest that prescription of sedative-hypnotic medications to treat insomnia is common, especially relative to insomnia diagnoses, and is likely the best proxy for identifying individuals with insomnia symptoms or insomnia disorder. Consults for insomnia-related concerns and documentation of CBT-I treatment sessions occurred less frequently than diagnoses or medication, though demonstrated an increasing trend over the course of the study period. Finally, group comparisons highlighted differences in insomnia indicators between individuals with varying sociodemographic and clinical characteristics. 

The current study builds upon existing work, such as that of Alexander et al. [6], by providing more recent estimates of the prevalence of insomnia-related diagnoses in US military veterans, albeit in a more focused sample (veterans receiving services within a regional network of VA healthcare facilities). As described earlier, Alexander et al. examined trends in the prevalence of insomnia diagnoses using national VA EHR data and identified that the age-adjusted annual prevalence of insomnia diagnoses ranged from 0.2% in FY2000 to 1.5% in FY2010 [6]. In the current study, the annual prevalence of insomnia diagnoses increased from 1.96% in FY2011 to 3.34% in FY2019. Thus, the increasing trend in annual prevalence of insomnia diagnoses observed by Alexander and colleagues was also observed in the current study. Despite the relative increases in insomnia diagnoses documented in medical records over time, there remains a notable discrepancy between estimates of insomnia derived from medical records (1.5–3.4% [6,7]) and those derived from epidemiology studies based on interviews and/or surveys (22–57% [9,22]). In both the current analysis and that of Alexander et al., relatively conservative criteria were used to define an insomnia diagnosis (i.e., ≥2 ICD codes occurring greater than 30 days apart but within 390 and 365 days, respectively). However, the discrepancy in prevalence estimates between medical record and interview- or survey-based studies is also apparent in studies that used medical record data but applied less stringent criteria for a diagnosis of insomnia (e.g., occurrence of a single insomnia diagnosis code, 7.4–11.8% [10]). Together, this suggests that insomnia is under-documented in the medical record and likely under-detected or under-diagnosed in clinical practice. 

In studies using data from the EHR, insomnia medication may dually signify the presence and treatment of insomnia disorder. Consistent with prior research [7,11], medications were the most frequent indicator of insomnia in the current study. The current findings add to prior research by identifying those with insomnia via a referral/consultation for the assessment/treatment of insomnia and engagement in CBT-I via standardized templated notes in the EHR. CBT-I had the lowest prevalence among the insomnia indicators and represented only ~9% of those with an insomnia ICD and only ~4% of those with an insomnia medication. This low prevalence could indicate a failure of providers to use the CBT-I-templated notes, indicative of low adherence to the EBP program’s policy of using standardized templated notes when delivering an EBP for which the VA has a training program (e.g., insomnia, depression, PTSD, etc.). This also potentially indicates a gap between the standard of care (i.e., CBT-I) and routine clinical practice (e.g., medication, sleep hygiene). Notably, however, though the first-line treatment for insomnia disorder, CBT-I is not universally effective or appropriate for all patients. Pharmacological treatments serve an important function in the management of insomnia, for instance, for those who do not respond to behavioral treatments, are ineligible (e.g., seizure disorder, significant substance use/withdrawal symptoms), or are otherwise unable to engage in behavioral treatment (e.g., work, caregiving barriers [23]). While the gap between the annual frequency of CBT-I and medication use remains large, the annual frequency of insomnia medications decreased over the course of the study period and the number of templated notes for CBT-I increased, perhaps representing a shift in routine care toward greater consistency with best practices for the management of insomnia disorder. 

By comparing sociodemographic and clinical groups, we also identified key group differences across the insomnia indicators. For example, Black veterans were significantly more likely than White veterans to have any insomnia indicator, and CONS/CBT-I appear to account for this difference; Black veterans were less likely to have an ICD, and there was no MED difference compared to White veterans. This is inconsistent with the literature suggesting that Black veterans engage less in psychotherapy than White veterans [24]. However, differences in the current analyses compared to the literature may be related to the disorder being treated (e.g., depression, trauma, insomnia, etc.). Similar findings are present for sex differences—female veterans had greater likelihood of CONS/CBT-I and lower likelihood of ICD compared to males, and females had slightly higher likelihood for MED. This finding was consistent with the literature regarding participation in psychotherapy between female and male veterans [25]. 

Other group differences were observed such that veterans with a mental health disorder or substance use disorder were less likely to engage in CBT-I, but more likely to receive an insomnia medication. For veterans with an active substance use disorder, this may be appropriate as CBT-I is not recommended for those with significant substance use and/or those experiencing withdrawal symptoms as these patients are more likely to have poor treatment attendance and poor adherence to treatment guidelines (e.g., stimulus control, sleep restriction). CBT-I may be appropriate for those with mild to moderate substance use or those in remission and/or no longer experience withdrawal symptoms. However, there is evidence that CBT-I is effective for those with comorbid mental health disorders (e.g., depression, PTSD) as well as evidence that treatment of insomnia may also benefit comorbid mental health conditions [14]. This finding is also consistent with previous VA EHR findings that veterans with a mental health disorder, especially PTSD, are less likely to be referred to for CBT-I [26]. 

Another large group difference occurred between veterans who live in non-metro versus metro counties. Veterans in non-metro counties were more likely to receive an insomnia diagnosis but much less likely to receive a consultation or engage in CBT-I. This low likelihood could be related to provider availability to deliver CBT-I at the smaller VA medical centers that are more likely to treat veterans who live in non-metro counties (i.e., Altoona, Butler, Coatesville, Erie, Lebanon, Wilkes-Barre vs. Philadelphia, Pittsburgh, Wilmington). This finding may also be due to differences in the process of engaging veterans in CBT-I—some VA medical centers in VISN 4 utilize a formal consultation in the EHR (i.e., the CONS variable in these analyses), notably the two largest (Philadelphia, Pittsburgh), and other VA medical centers do not, relying instead on consultations to a mental health clinic or perhaps informal referrals to CBT-I providers that are not documented in the EHR (e.g., email, phone calls). 

### 4.1. Limitations 

EHR data allow for naturalistic observations of practices and procedures used in routine clinical care across a large number of settings, providers, and patients. The primary limitation of analyzing data from EHR, however, is that the data are limited to what is input by providers. Despite efforts to standardize aspects of data input into EHR, there will always be variability and inaccuracy. As noted above, the documentation of the delivery of CBT-I is supposed to utilize the national templated notes for CBT-I developed by the VA Office of Mental Health and Suicide Prevention EBP program. It was beyond the scope of this study to try and identify and confirm delivery of CBT-I with non-templated notes. However, non-templated progress notes are often used to document delivery of EBPs for insomnia and other treatments (e.g., depression, trauma, anxiety, SUD, etc.). Furthermore, delivery of CBT-I in our analyses is underestimated as templated notes were only available for part of our study period (FY2015–2019). A similar issue of underestimation may also be present for sedative-hypnotic medications. The list we used was selected in part due to the high level of confidence that the specific drugs in specific dose ranges were used to treat insomnia; however, numerous other drugs treat insomnia but there is greater likelihood of their use for non-insomnia disorders/symptoms (e.g., sedating anti-depressants, benzodiazepines, anti-psychotics). Still, we found similar rates of sleep medications as a FY2010 national veteran dataset that reported 13.7% of veterans were prescribed an anxiolytic or hypnotic (primary indication per the Food and Drug Administration, except for trazodone which was reclassified as a hypnotic; [7]). In sum, data for this study and similar EHR analyses are based on clinician input and the associated variation and lack of standardization. 

Next, it is important to consider that documentation in the EHR is simply that and does not reflect severity. An ICD code or prescription medication does not indicate if the insomnia is mild or severe, only present. Clinical interviews and/or the use of established self-report measures would help to confirm the level of severity of insomnia among veterans. The CBT-I-templated note can help as this includes an initial evaluation note, which consists of a clinical interview by a trained provider. Another way to assess insomnia severity is with the Insomnia Severity Index (ISI), a well-established self-report measure that is often delivered to veterans but may be inconsistently uploaded into the EHR. While the ISI is a component of the CBT-I-templated notes, extracting the ISI information from the notes was beyond the scope of the current study but will be pursued in future analyses. Further, with these data we are unable to determine whether VA is the primary source of medical and mental health care for a given individual; to be included in the sample, veterans needed >1 visit, but non-VA data was not available for analysis. It is possible that individuals who seek both VA and non-VA care were diagnosed with and/or received treatment for insomnia outside of the VA. 

### 4.2. Future Directions

This study highlights the continued need for efforts to support the detection of insomnia and management using first-line treatments, such as CBT-I. Identified group differences may help to serve as targets for improving efforts for the assessment/treatment of insomnia, and greater attention should be paid to differences in insomnia care based on sex, race, rurality, and clinical comorbidities. Extending this current line of research, it would be informative to identify whether these and other factors are related to patient participation, or non-participation, in CBT-I. Continued efforts to disseminate CBT-I training to improve access to care and further engage veterans in the recommended treatment for insomnia will also be valuable. Finally, though the current study focuses on insomnia and insomnia-related care, it is important to also acknowledge the value of examining how current models of care address sleep health more generally. Sleep health refers to “a multidimensional pattern of sleep-wakefulness, adapted to individual, social, and environmental demands, that promotes physical and mental well-being” and “is characterized by subjective satisfaction, appropriate timing, adequate duration, high efficiency, and sustained alertness during waking hours” [27] (p. 12). In addition to investing in the roll-out of CBT-I, the VA is also committed to furthering models of care that support sleep health, such as the Whole Health approach. The Whole Health approach represents a person-centered and holistic model of care. “Recharge” is one of the eight areas of self-care within the Whole Health model and includes a focus on sleep and rest to improve physical and emotional health. Future studies may use VA EHR data to examine how the Whole Health approach affects sleep health in the veteran population and how sleep health is addressed in the VA model of care. Additional planned analyses involving the current cohort data will involve sociodemographic variables (race, rurality, sex) and specific diagnoses and combinations of diagnoses (e.g., depression, PTSD, chronic pain, heart disease) on the impact of engagement in treatment (i.e., CBT-I, medication, neither).

## 5. Conclusions

This study suggests that in a regional VA network, the prevalence of insomnia diagnoses is increasing but remains less common than prescription of sedative-hypnotic medications used to treat insomnia. Aligned with VA’s investment in the roll-out of CBT-I, consults for insomnia-related concerns and documentation of CBT-I treatment sessions have increased since 2011. Still, these are significant gaps between insomnia indicators in the VA EHR that are not consistent with clinical practice guidelines. Continued efforts are necessary to improve VA’s recognition, and documentation, of insomnia and subsequent treatment with CBT-I over medications as the standard of care. Additional analyses of VA EHR data will help to identify groups to target to ensure care is equitable and consistent with clinical practice guidelines. VA remains the leader in delivering insomnia-related care and further evaluation of EHR data can help to improve care and improve veteran health and quality of life.

## Figures and Tables

**Table 1:** Prevalence and count of insomnia indicators by year.

Full Sample		ICD		MED		CONS		CBTI	
Year	*n*	%	*n*	%	*n*	%	*n*	%	*n*
2011	245,833	1.96%	4829	9.08%	22,313	0.14%	338	--	--
2012	258,349	2.45%	6342	8.95%	23,131	0.16%	412	--	--
2013	257,814	2.59%	6668	8.89%	22,926	0.19%	490	--	--
2014	258,479	2.75%	7108	8.73%	22,553	0.24%	630	--	--
2015	258,538	2.89%	7471	8.62%	22,282	0.36%	934	0.12%	300
2016	258,076	2.69%	6943	8.41%	21,702	0.39%	996	0.19%	488
2017	258,739	2.93%	7593	7.99%	20,675	0.32%	838	0.19%	486
2018	259,948	3.10%	8048	7.59%	19,718	0.46%	1188	0.21%	547
2019	260,033	3.34%	8677	7.32%	19,042	0.54%	1392	0.24%	620

**Table 2:** Insomnia indicators by age (young vs. old; deciles).

	Any Insomnia		ICD		MED		CON		CBTI	
	%	*n*	%	*n*	%	*n*	%	*n*	%	*n*
Age	17.04%	74,966	6.00%	26,375	14.56%	64,036	1.51%	6627	0.55%	2441
<50	25.39%	28,268	9.33%	10,393	21.23%	23,634	3.03%	3375	1.09%	1210
≥50	14.21%	46,698	4.86%	15,982	12.30%	40,402	0.99%	3252	0.37%	1231
20	25.70%	10,535	10.05%	4118	20.66%	8468	3.41%	1397	1.07%	439
30	26.05%	7180	9.45%	2603	21.99%	6060	2.95%	813	1.07%	296
40	24.66%	10,553	8.58%	3672	21.28%	9106	2.72%	1165	1.11%	475
50	23.40%	15,516	7.71%	5110	20.73%	13,745	2.07%	1374	0.84%	559
60	16.18%	20,375	5.47%	6883	14.05%	17,689	1.14%	1430	0.46%	578
70	8.32%	6086	3.06%	2240	6.94%	5081	0.39%	289	0.10%	73
80	7.49%	4327	2.78%	1608	6.16%	3560	0.26%	150	0.04%	21
90	7.30%	394	2.61%	141	6.06%	327	0.17%	9	0.00%	0

**Table 3:** Prevalence and relative risk of veterans with any insomnia and individual insomnia indicators.

	Any Insomnia ^a^ (*n* = 74,966)			ICD ^b^ (*n* = 26,375)			MED ^b^ (*n* = 64,036)			CONS ^b^ (*n* = 6627)			CBTI ^b^ (*n* = 2441)		
	%	RR	95% CI	%	RR	95% CI	%	RR	95% CI	%	RR	95% CI	%	RR	95% CI
Female	26.5	1.61	1.58–1.65	33.5	0.95	0.92–0.98	87.3	1.02	1.01–1.03	9.9	1.13	1.05–1.22	4.5	1.44	1.29–1.62
Male	16.4			35.4			85.2			8.7			3.1		
Black	24.3	1.47	1.45–1.49	32.4	0.90	0.88–0.93	85.4 *	1.00	0.99–1.01 *	12.4	1.54	1.46–1.62	5.1	1.82	1.67–1.99
Other	11.3	0.69	0.67–0.71	35.6 *	0.99	0.95–1.04 *	83.9	0.98	0.97–1.00 *	8.5 *	1.06	0.96–1.17 *	3.6	1.29	1.10–1.52
White ^c^	16.5			35.8			85.5			8.0			2.8		
<50	25.4	1.79	1.76–1.81	36.8	0.92	0.91–0.94	83.6	0.97	0.96–0.97	11.9	1.72	1.64–1.80	4.3	1.62	1.50–1.76
≥50	14.2			34.2			86.5			7.0			2.6		
MH	32.6	4.48	4.41–4.55	33.4	0.83	0.81–0.85	88.4	1.15	1.14–1.16	8.5	0.88	0.83–0.92	3.1	0.80	0.73–0.87
No MH	7.3			40.3			76.9			9.7			3.8		
SUD	30.1	2.13	2.10–2.16	32.0	0.87	0.85–0.89	90.0	1.08	1.08–1.09	8.1	0.88	0.84–0.93	2.3	0.63	0.57–0.69
No SUD	14.1			36.7			83.2			9.2			3.7		
Non-Metro	15.0	0.88	0.83–0.92	39.7	1.13	1.05–1.22	84.2 *	0.99	0.96–1.01 *	5.5	0.62	0.49–0.79	1.0	0.29	0.16–0.52
Metro	17.1			35.1			85.4			8.9			3.3		

Notes: ^a^ Comparison of the Any Insomnia group (any of ICD, MED, CONS, CBTI) vs. No Insomnia group; ^b^ comparison within the Any Insomnia group for those with vs. without the specific indicator: ICD, MED, CONS, or CBTI; ^c^ White race is the reference group for Black and Other race comparisons; all *p*-values < 0.01 unless indicated (* not significant); MH, mental health disorder; SUD, substance use disorder.

## Data Availability

No new data were created or analyzed in this study; all data were retrospective from electronic health records. Data sharing is not applicable to this article.
